# Determining the ideal measurement site and respiratory condition for liver transient elastography: toward clinical practice standardization

**DOI:** 10.1186/s13244-024-01692-x

**Published:** 2024-05-12

**Authors:** Zihao Huang, Sai Kit Lam, Lok Kan Cheng, Yangmin Lin, Yongping Zheng

**Affiliations:** 1 Department of Biomedical Engineering, The Hong Kong Polytechnic University, Hong Kong, China; 2 Research Institute for Smart Ageing, The Hong Kong Polytechnic University, Hong Kong, China

**Keywords:** Liver elastography, Ultrasound elastography, Intercostal imaging, Measurement protocol, Measurement guideline

## Abstract

**Objectives:**

Liver transient elastography (TE) has been endorsed by the WHO as the first-line diagnostic tool for liver diseases. Although unreliable and invalid results caused by intercostal space (ICS)-associated factors (including excessive subcutaneous fat and a narrow ICS relative to the transducer size) and operator inexperience are not uncommon, no standard guidelines for ideal probe placement are currently available. Herein, we conducted a prospective observational study to identify an ideal measurement site and respiratory condition for TE by characterizing anatomical and biomechanical properties of the ICSs using ultrasound B-mode and elasticity imaging.

**Methods:**

Intercostal ultrasound was performed pointwise at four specific sites in 59 patients to simultaneously measure the width, stiffness, and skin‒liver capsule distance (SCD) of the ICSs over the liver, under end-inspiratory and end-expiratory conditions. Intersections between the 8th ICS and anterior axillary line, the 7th ICS and anterior axillary line, the 8th ICS and mid-axillary line, and the 7th ICS and mid-axillary line were defined as Sites 1 to 4, respectively.

**Results:**

Results indicated that Sites 2 and 3 presented greater intercostal width; Sites 3 and 4 displayed lower intercostal stiffness; Sites 2 and 3 exhibited a shorter SCD. The ICSs were significantly wider and stiffer at end-inspiration. Additionally, the liver was more easily visualized at Sites 1 and 3.

**Conclusion:**

We recommend Site 3 for TE probe placement owing to its greater width, lower stiffness, and smaller abdominal wall thickness. Performing TE at end-inspiration is preferred to minimize transducer-rib interferences. This study paves the way toward a standardized TE examination procedure.

**Critical relevance statement:**

A standardized measurement protocol for WHO-recommended liver TE was first established to improve the success and efficiency of the examination procedure.

**Key Points:**

- WHO-recommended TE is unreliable or fails due to intercostal space-related factors.
- The 8th intercostal space on the mid-axillary line and end-inspiration are recommended.
- This standardized protocol aids in handling challenging cases and simplifies operational procedures.

**Graphical Abstract:**

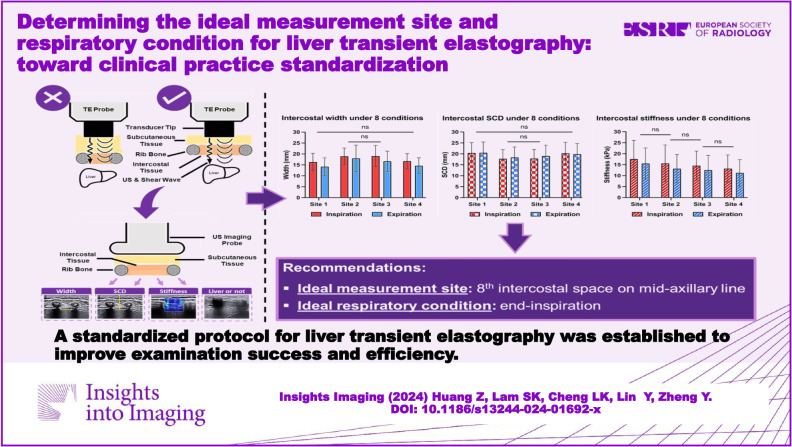

## Introduction

The severity of liver fibrosis is linked with all-cause and liver-related mortality, necessitating early diagnosis and continuous monitoring [1]. Transient Elastography (TE) is an emerging technological advance in ultrasound (US) imaging for assessing the presence and degree of liver fibrosis via stiffness measurement [2–6]. Its superior features of being noninvasive, easy to operate, low cost, rapid, and reliable, having high patient compliance, as well as being proven to offer histopathological correlations have provided the community with a favorable alternative compared to the current clinical standard of liver biopsy. According to major clinical guidelines [3, 4, 7, 8] and the WHO [9, 10], TE has been endorsed as the first-line assessment for chronic liver diseases [11], and its popularity is on a rapid rise in the field of hepatology globally.

Nevertheless, the factors affecting the harvest of reliable and valid examination results during the implementation of the TE technique remain incompletely elucidated. During a TE procedure, an operator places a cylinder-like single-element transducer at a location between the ribs, in which the transmission of both ultrasound and shear waves take place from the skin surface through the intercostal space (ICS) and its overlying abdominal wall layers, and ultimately into liver parenchyma for liver stiffness measurement. Following this principle, a successful TE examination relies heavily on the morphological and biomechanical characteristics of the ICS where the ultrasound probe is placed, which may in concert, influence the effectiveness of wave propagation.

In the current body of literature, there is accumulating evidence regarding the deficiencies of the TE examination when it comes to patients with narrow ICS [2, 12, 13] or obesity [2, 13–15] and for operators with inadequate experience [14, 15]. A transducer suboptimally positioned interferes with the ICS configuration in lean and obese patients, even in normal-BMI individuals. Lean individuals with limited rib spacing (i.e., narrow ICS) may experience invalid mechanical excitation of shear waves primarily at rib bones instead of intercostal tissues, resulting in minimal wave generation. In addition, the presence of ribs is prone to de-focalization of the traveling waves, leading to complex wave propagation patterns and miscalculations of results. On the other hand, obese individuals present excessive subcutaneous adipose accumulation, severely attenuating the waves before reaching the liver. Apart from specific patient subpopulations, TE results could be difficult to obtain from normal-BMI patients due to operator inexperience (i.e., operation on < 500 examinations) [14, 15]. In the largest longitudinal study to date [14], a French team examining 7261 patients over a 5-year period, reported that the frequency of failure and unreliable TE results could reach as high as 3.1 and 15.8%, respectively. Similar results have been reported in another Asian cohort (*n* = 3205), with failures observed in 2.7% of cases and unreliable results in 11.6% [16]. In light of the above evidence, there is an urgent demand in the community for guiding operators in the placing of the transducer, for the sake of mitigating failure or unreliable rate of TE and ultimately maximizing its efficacy in the general population.

**Fig. 1:**
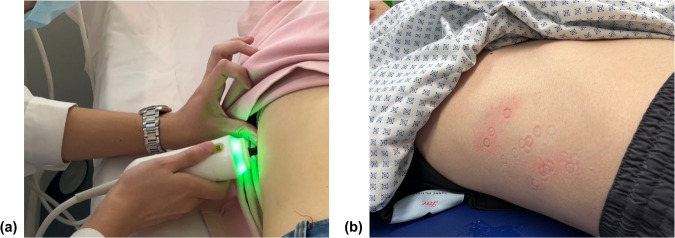
TE examination. **a** Setup of the placement of the TE probe in an ICS; **b** a representative patient in real-world practice, who was undergoing the TE examination using a trial-and-error method due to the absence of clear guidelines for selecting a measurement site. Each red mark on the patient’s skin surface corresponds to a single measurement attempt made using the transducer tip

There is no existing guideline on the selection of measurement sites for a standard practice in this regard [17]. Under this circumstance, operators could only rely on their own experience or a trial-and-error method during the procedure, which may largely reduce examination efficiency as well as patient comfort (Fig. 1). On top of that, there is no research on the potential respiratory effects on the TE examination procedure. All things considered, it is imperative to investigate the anatomical characteristics of those commonly used ICSs over the liver, and specify an ideal probe placement condition for TE practitioners.

The overarching goal of this observational study was to identify an ideal site and respiratory condition for TE probe placement, through the investigation of the anatomical and biomechanical characteristics of the ICSs on the human right inferior rib cage using a combination of shear-wave elastography (2D-SWE) and B-mode imaging. Specifically, the primary objective of this cross-sectional study was to assess the differences in intercostal width, stiffness, and skin‒liver capsule distance (SCD) among the selected ICSs under different respiratory conditions. We hypothesized that intercostal anatomy varied within individuals, and at least one ideal site would stand out to favor TE. The secondary objective was to analyze the factors associated with intercostal width and stiffness, and examine the anatomical relationship of the ICSs with the presence of the liver.

## Methods

### Study population

In this prospective study, adults (≥ 18 years) with known chronic liver diseases of any etiology who had a clinical indication for TE were eligible. The sample size of 62 was estimated using the G*Power software [18], assuming a median effect size of 0.15, statistical power of 0.95, and a two-sided significance level of 0.05 under a repeated-measures design. Exclusion criteria consisted of chest wall deformity or a history of previous thoracic or abdominal surgery. This observational cross-sectional study was designed, conducted, and reported in accordance with the STROBE statement [19]. All subjects provided written informed consent prior to enrolment. The research was approved by the institutional review board of The Hong Kong Polytechnic University (HSEARS20210809002), and conformed to the Declaration of Helsinki.

### Procedures

**Fig. 2:**
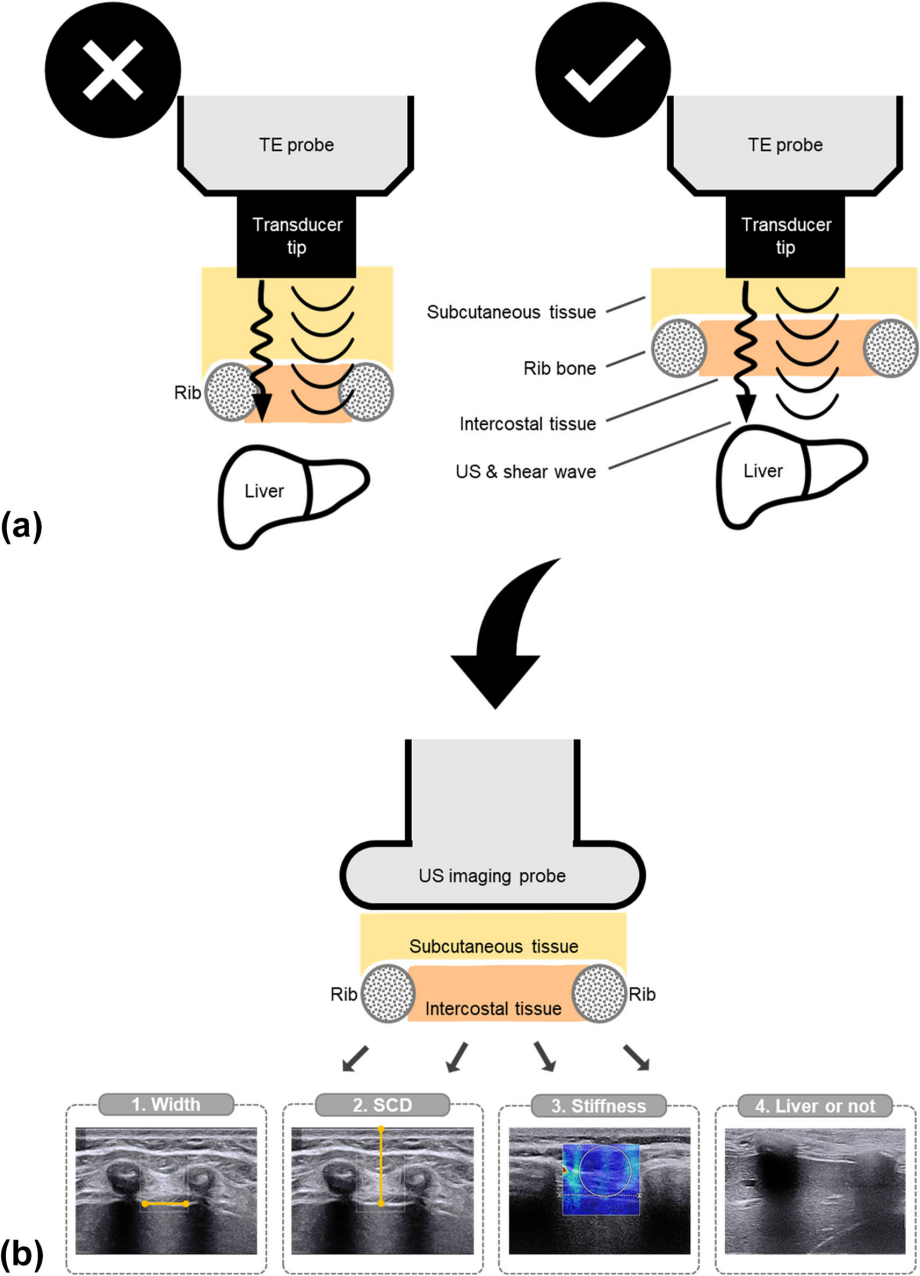
Study overview. **a** Clinical problem: the impact of the ICS configuration on the transmission of shear waves into the liver and the ultrasound used to measure their speed of propagation during the TE examination; the left-hand side of the figure illustrates a failed case, where the transducer is placed on a thick layer of subcutaneous tissue and a narrow ICS; the right-hand side of the figure depicts an ideal situation, where the transducer is placed on an appropriate thickness of subcutaneous tissue and a wide ICS; **b** research methodology: anatomical characterization of the ICS via US measurements of (1) intercostal width, (2) SCD, (3) stiffness, and (4) the detection of the presence of the liver

The overall methodology is depicted in Fig. 2. Intercostal 2D US imaging was performed using a linear probe (SL15-4, central frequency: 8.5 MHz) of the Aixplorer® system (SuperSonic Imagine, Aix-en-Provence, France) which enables simultaneous B-mode imaging and stiffness mapping. Subjects were positioned in the supine posture and raised their right arm maximally, adhering to the standard patient positioning used for liver TE examination [2].

**Fig. 3:**
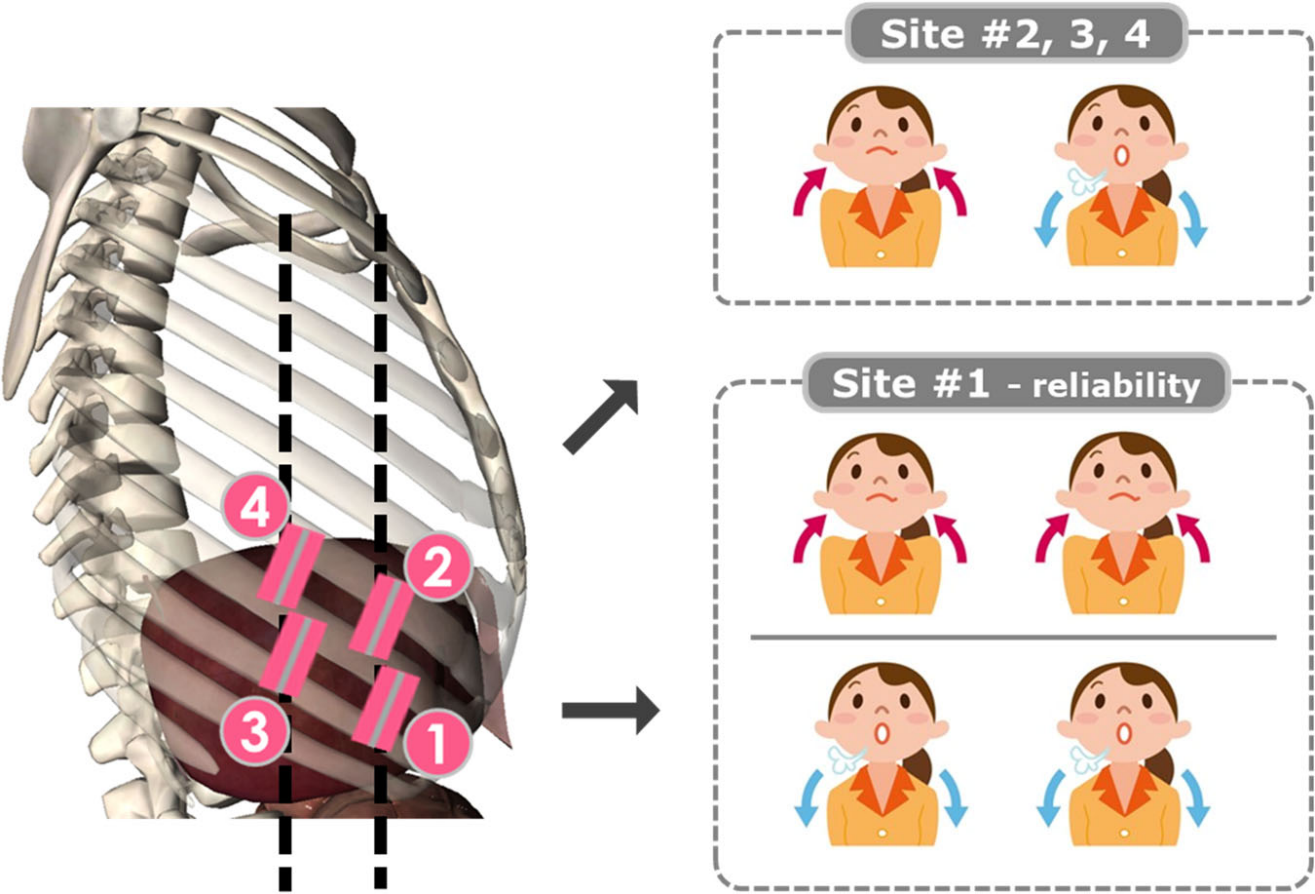
Experimental protocol. Selection of the four measurement sites and the two respiratory conditions

Four sites of interest in the right inferior part of the rib cage over the liver were selected to investigate a hypothesized inter-ICS difference. The selection of these four sites was referenced from the locations, which were reported in the previous liver ultrasound elastography-related literature and may be commonly used in clinical practice [17, 20–24]. The investigator located and marked the sites using the following standardized anatomical landmarks: Site 1 (intersection between the 8th ICS and anterior axillary line); Site 2 (intersection between the 7th ICS and anterior axillary line); Site 3 (intersection between the 8th ICS and mid-axillary line); Site 4 (intersection between the 7th ICS and mid-axillary line). Each subject underwent intercostal US at each of the four sites under two respiratory conditions: (a) end-inspiration and (b) end-expiration (Fig. 3), resulting in a total of eight experimental conditions. These conditions were executed as a transient breath-hold during normal breathing. The probe was placed with minimal pressure at the skin surface of each site. The probe orientation was fine-tuned around the marked site, until its width was estimated to be minimal, indicating that the probe was perpendicularly oriented to the ribs. A 6-s video, comprising sequential images of one half-respiratory cycle, was acquired from the eight experimental conditions. A linear probe (SL15-4, central frequency: 8.5 MHz) was also employed to observe the presence of the liver underneath each site. Settings for depth and time-gain compensation (TGC) were fined-tuned to achieve a deeper field of view of the underlying organ. A binary criterion (i.e., presence or absence) was utilized to determine whether a liver parenchymal portion with at least 4 cm thickness could be visualized in the cross-sectional B-mode images.

### Outcome measures

#### B-mode-derived intercostal width and SCD

**Fig. 4:**
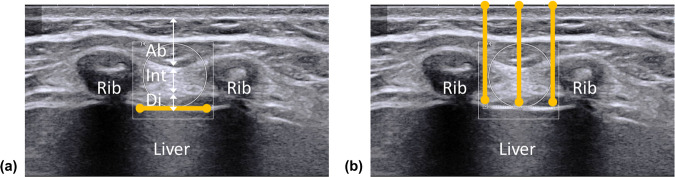
Illustration of morphological imaging of the ICSs. **a** Width and (**b**) SCD measurements in the B-mode image of the ICSs (Ab: abdominal muscles; Int: intercostal muscles; Di: diaphragm muscle)

The width of the ICS was measured to examine its geometric relationship with the size of a TE transducer. It was defined as the horizontal length between the inferior border of the upper rib and the superior border of the lower rib, where acoustic shadowing caused by rib bones is widest (Fig. 4a). Linear measurements of the width were consistently taken at the level of an innermost echogenic layer of the diaphragmatic border. The SCD of the ICS corresponds to abdominal wall thickness, which was measured from the skin surface to the pleural line (Fig. 4b). Due to the varying shape of intercostal tissues, the SCD value is not necessarily the same within one ICS. Thus, we repeated SCD measurements at three different locations within the ICS (i.e., its center, left-most boundary, and right-most boundary). These three measurements were then averaged, which became the representative SCD for that ICS.

#### SWE-derived intercostal stiffness

**Fig. 5:**
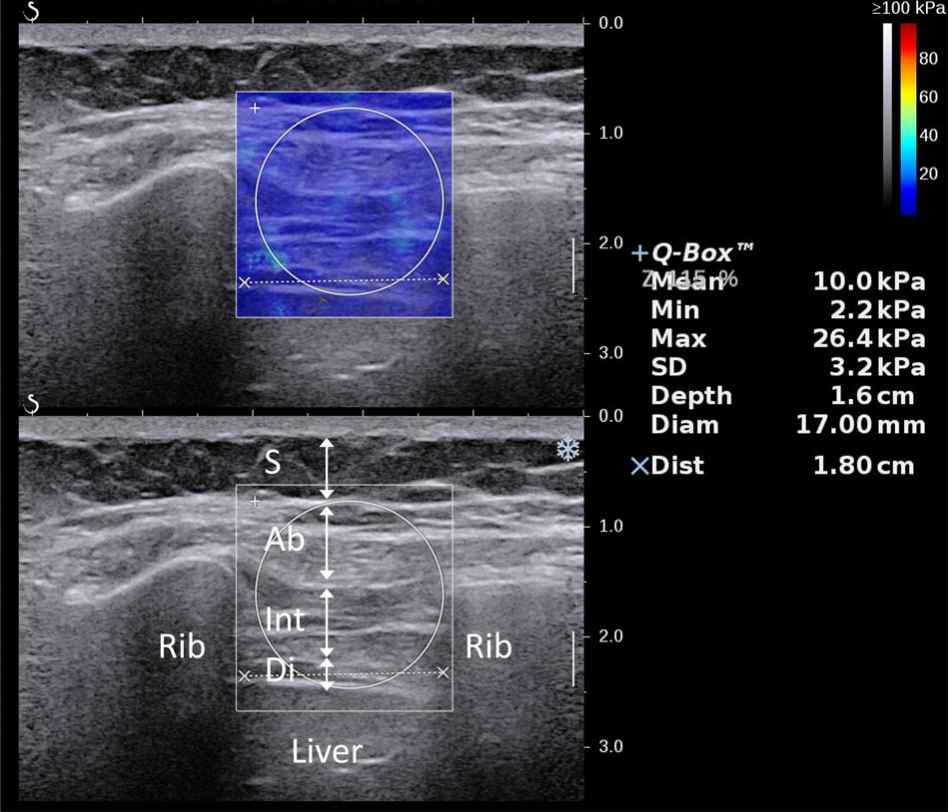
Illustration of elasticity imaging of the ICSs. Stiffness measurement in the SWE image of the ICSs. (S: subcutaneous fat; Ab: abdominal muscles; Int: intercostal muscles; Di: diaphragm muscle)

During the SWE acquisition process, an SWE box was placed at the center of an ICS and customized to include the cross-sectional area of the external oblique, intercostal muscles, diaphragm, and portions of ribs and subcutaneous fat (Fig. 5). Intercostal stiffness represents the axial strain of the ICS in response to the probe pressure. This biomechanical parameter assesses the degree to which the ICS is accommodating to the placed transducer tip.

We further analyzed the intra- and inter-observer reliability of the intercostal width, SCD, and stiffness measurement, respectively. The protocol is detailed in the Supplementary Material.

### Data analyses

Image post-processing was performed to quantify the width, stiffness, and SCD of the ICS in the intercostal US images using a dedicated workstation provided by the manufacturer. The measurement data were collected by the third-party rater, who was blinded to the actual experimental condition of the images being analyzed. Details of these quantification procedures are described in the Supplementary Material.

### Statistical analyses

#### Descriptive statistics

Cohort characteristics were descriptively summarized and reported as medians (IQRs) for continuous variables and proportions (frequencies) for categorical variables. Two-sided *p* values less than 0.05 were considered statistically significant.

#### Effect of respiration and site on US measurement

Two-way repeated measures ANOVAs were conducted to analyze the main and interaction effects of two factors: (a) ‘respiration’ factor (end-inspiratory vs. end-expiratory conditions) and (b) ‘measurement site’ factor (Site 1, 2, 3, 4) on the US-derived width, stiffness and SCD of the ICSs, respectively.

#### Likelihood of visualizing the liver underneath sites

Cochran’s Q test was applied to test the differences in detecting the presence of the liver across the sites.

## Results

### Subject demographics

Between May and December 2022, 62 consecutive adult patients with known chronic liver diseases were recruited in the study. Three (4.8%) subjects were excluded due to withdrawal of consent (*n* = 2) and accidental data loss (*n* = 1). The characteristics of the remaining 59 subjects included in the analyses are summarized in Table 1; the aggregated anatomical data for each site of interest are presented for comparison.

**Table 1:** Characteristics of the study cohort (*n* = 59)

Variable	Median [IQR] or proportion % (*n*)
•Demographics	
Male gender	49% (29)
Age, years	57 [43–64; range 27–71]
Overweight & obese	34% (20)
Metabolic syndrome	12% (7)
•Anthropometrics	
BMI, kg/m^2^	24 [21–26; range 1–39]
Waist circumference, cm	87 [79–94; range 66–115]
•ICS morphology	
Width at Site 1, mm	14.9 [12–18; range 6–26]
Width at Site 2, mm	18.0 [15–21; range 8v53]
Width at Site 3, mm	17.4 [14–21; range 5–32]
Width at Site 4, mm	15.4 [13–18; range 7–25]
SCD at Site 1, mm	19.3 [18–23; range 13–39]
SCD at Site 2, mm	16.6 [15–21; range 11–38]
SCD at Site 3, mm	17.6 [15–21; range 11–39]
SCD at Site 4, mm	19.6 [16–23; range 10–37]
•ICS biomechanics	
Stiffness at Site 1, kPa	15.1 [11–20; range 6–51]
Stiffness at Site 2, kPa	12.6 [9–17; range 4–46]
Stiffness at Site 3, kPa	11.7 [9–16; range 5–48]
Stiffness at Site 4, kPa	10.0 [8–14; range 4–40]

Data were aggregated for two respiratory conditions

*BMI* body mass index

### Reliability of US measurement

Intercostal width and SCD measurements are highly reproducible irrespective of respiratory conditions (ICC ≥ 0.97), while intercostal stiffness measurement demonstrated fair to good reliability (interoperator ICC_(2, 3)_: 0.593–0.683; intraoperator ICC_(3, 3)_: 0.790–0.896, see Supplementary Table 1).

### Comparison of respiration and site in US measurement

#### Intercostal width measurement

**Fig. 6:**
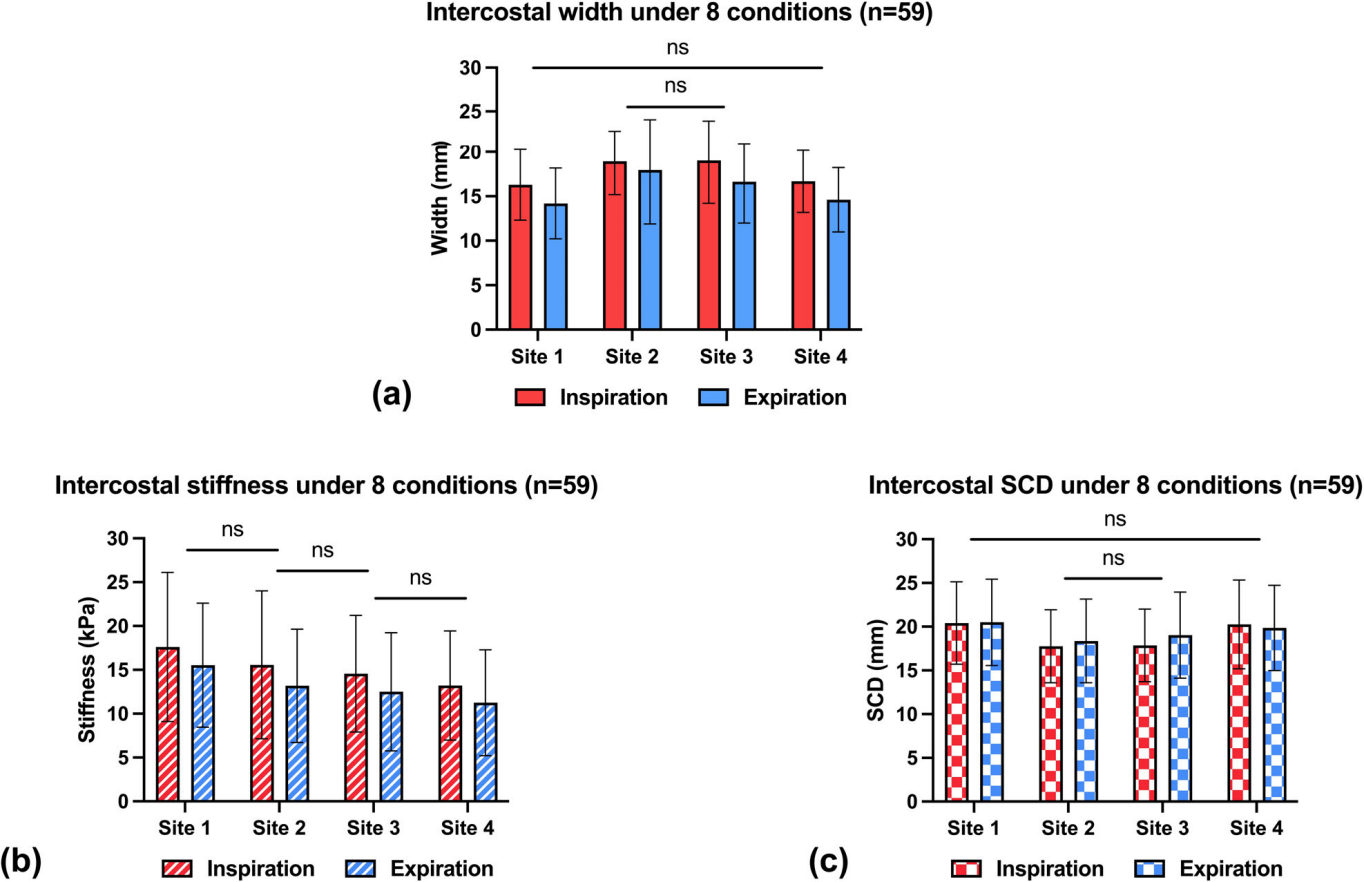
Morphological and biomechanical data of the ICSs. US-derived (**a**) width, (**b**) stiffness, and (**c**) SCD of the ICS under eight experimental conditions (ns: *p* > 0.05; *p* ≤ 0.05 otherwise)

No significant interaction effect was found between the factor of ‘site’ and ‘respiration’ on intercostal width (*F* [1, 80] = 3.07; *p* = 0.07). The results showed a significant main effect of ‘respiration’ (*F* [1, 58] = 75.27; *p* < 0.001), with inspiration producing greater intercostal width values (Fig. 6a). The main effect of ‘site’ was also significant (*F* [2, 129] = 17.25; *p* < 0.001). Post hoc comparisons among all sites revealed that the ICSs at Site 2 (18.4 mm, 95% CI: 17.3–19.6) and Site 3 (17.8 mm, 95% CI: 16.6–19.0) were significantly wider than those at Site 1 (15.2 mm, 95% CI: 14.2–16.2) and Site 4 (15.6 mm, 95% CI: 14.7–16.5).

#### Intercostal stiffness measurement

Similarly, although there was no two-way interaction between two factors (*F* [2, 138] = 0.05; *p* = 0.967), the significant main effects of ‘respiration’ (*F* [1, 58] = 15.24; *p* < 0.001) and ‘site’ (*F* [3, 161] = 10.78; *p* < 0.001) factors were identified on the stiffness values (Fig. 6b). Respiratory movement generally increased intercostal stiffness from the end-expiratory to end-inspiratory phase. Intercostal stiffness was significantly lower at Site 4 (12.2 kPa, 95% CI: 10.7–13.7) than at Site 1 (16.6 kPa, 95% CI: 14.9–18.3) and Site 2 (14.4 kPa, 95% CI: 12.7–16.1). The intercostal stiffness values of Sites 3 and 4 were observed to be similar (a mean difference of 1.3 kPa, *p* = 0.335), irrespective of respiratory conditions.

#### Intercostal SCD measurement

An interaction effect between ‘site’ and ‘respiration’ existed in explaining the difference in intercostal SCD (Fig. 6c), *F* [3, 174] = 9.75; *p* < 0.001. This implied that the effect of respiration is site-dependent. Therefore, simple main effects of ‘site’ were run separately for the end-inspiratory and end-expiratory conditions. While Site 2 (17.8 ± 4.2 mm) and Site 3 (17.9 ± 4.1 mm) shared significantly shorter end-inspiratory SCDs, Site 2 (18.4 ± 4.8 mm) exhibited the shortest end-expiratory SCD.

#### Influencing factors on intercostal width and stiffness

Detailed analyses are presented in Supplementary Table 2 and Supplementary Table 3.

#### Difference in liver detection rates

Table 2 presents the relationship between the site and the presence of the liver under the two respiratory conditions. Cochran’s Q test determined statistically different proportions among the four sites below which the liver is visible at end-inspiration, χ^2^(3) = 44.357, *p* < 0.001. The post hoc Dunn’s test found that the liver was significantly better visualized at Sites 1 and 3 (100% and 90%, *p* = 0.989) than Sites 2 and 4. On the other hand, the test did not indicate any differences among the four proportions during the end-expiratory phase, χ^2^(3) = 0.857, *p* = 0.836.

**Table 2:** Liver detection rates across four measurement sites under two respiratory conditions (*n* = 59)

	Is the liver the underlying organ visible in B-mode images?			
	7th ICS		8th ICS	
	Site 2	Site 4	Site 1	Site 3
End-inspiration^a^	44 (75%)	32 (54%)	59 (100%)	53 (90%)
End-expiration^b^	56 (95%)	56 (95%)	55 (93%)	57 (97%)

Data were presented as n (%)

^a^*p* > 0.05 for Site 1 vs. Site 3, Site 2 vs. Site 3; *p* ≤ 0.05 for remaining pairs otherwise

^b^*p* > 0.05 for all pairs

## Discussion

A right lateral intercostal approach is commonly used in the TE examination procedure to access the liver. Understanding the anatomical relationship between the ICS and transducer is of clinical importance, as it influences the success and efficiency of the examination. Knowledge of intercostal anatomy can assist in managing transducer‒rib interferences, but is lacking in the literature. Hence, this US-based observational study sought to establish evidence-based recommendations for a TE measurement protocol, including a specific measurement site and respiratory condition. It is the first work towards the standardization of the TE examination procedure, ultimately maximizing its effectiveness for liver disease patients.

### Intercostal anatomy and implication for site choice

To better accommodate the transducer tip and mitigate interferences from adjacent ribs and overlying fat layers, we anticipated the ICS to be wider, thinner, and softer. Our anthropometry analyses revealed that the anatomical characteristics of the ICSs are site-dependent, with significantly greater intercostal width at Sites 2 and 3, shorter intercostal SCD at Sites 2 and 3, and lower intercostal stiffness at Sites 3 and 4. Previous studies on ICS anatomy are scarce, with limited published data available concerning intercostal width over the liver region. Only one related work carried out by Kim et al [25] showed substantial variation in the widths of the six ICSs on the right inferior rib cage, with the 7th ICSs on the anterior axillary line being the widest (mean width of 18.3 ± 3.4 mm). Notably, this reported width closely matched our data for that particular ICS (Site 2: mean width of 18.1 ± 4.5 mm; median width of 18.0 mm with IQR 5.8 mm). Moreover, our demographic analysis revealed that 6.8% and 44.1% of the subjects in our cohort had intercostal width values smaller than 9 mm (diameter of the M probe tip for standard morphotype [2]) and 12 mm (diameter of the XL probe tip for obesity [26]), respectively. These relatively narrow ICSs might partly account for the previously reported TE failure or unreliable results in the literature. This finding further emphasizes the importance of considering intercostal dimensions when designing future transducer specifications. Pradhan et al [12] were also aware of the limitation of TE in small adults who frequently have narrow ICSs; they proposed a solution to utilize the S probe, which configures a smaller 7-mm diameter tip to minimize rib interference. In contrast, our study introduced a more universal and adaptive approach to the community by providing TE practitioners with an ideal probe placement site, even for patients of small stature with generally narrower ICSs. This effectively waives the need to modify the type of TE probe in practice. Intercostal elastography also remains an unexplored territory in the literature. Only two recent studies [27, 28] demonstrated the feasibility of biomechanical characterization of the ICS by SWE. The reported biomechanical values (an approximation of 10 to 20 kPa) were of the same order of magnitude as our data, which supports the validity of our intercostal SWE results. However, those studies were limited to measuring only one representative ICS in the adolescent scoliosis cohort. In contrast, our study presented a distinct research question and was the first attempt to investigate the biomechanical differences among multiple ICSs in adults. Additionally, abdominal wall thickness is the determinant of TE failure, as it relates to the hindrance of propagation of ultrasound and shear waves [26]. To adapt to a greater amount of subcutaneous tissues in obese patients, the XL probe with a lower ultrasound frequency and a higher vibration amplitude was specifically designed [6, 26]. Our study identified two specific ICSs with relatively thinner abdominal walls, which may provide another insight into addressing the overweight population, particularly when an XL probe is not available. In summary, Site 3 consistently emerged as the superior site for TE probe placement, given its greater width, lower stiffness, and shorter SCD.

### Respiratory effect on intercostal anatomy

End-inspiration appeared to consistently yield the wider and stiffer ICSs across all measurement sites in the present study. We speculated that an increased thoracic perimeter and inspiratory muscle activation during inhalation played a role. The finding is consistent with the previous studies that reported an increase in intercostal width [27], stiffness [27–29], muscle cross-sectional area [30], and muscle thickness [31] during inspiration. On the other hand, the impact of respiration on the intercostal SCD values exhibited a non-linear pattern, the ANOVA suggesting a site-dependent interaction. This observation implies that respiratory movement behaved differently at various sites. Factors such as lung volume, intercostal muscle recruitment, and pleural pressure collectively contribute to the overall shape of intercostal tissues [32], influencing the derived SCD.

### Presence of the liver and implication for site choice

Compared to the 7th ICS, there seemed to be a higher likelihood of visualizing the liver via the 8th ICS. In the 7th ICS, the frequency of occurrence of the liver was lower at end-inspiration, whereas no such trend was observed in the 8th ICS. This phenomenon can be explained by lung inflation during the inspiratory phase, causing the liver to move cranio-caudally. Consequently, the upper ICSs are more likely to present in the lung rather than the liver. Our analysis was not attempting to describe a comprehensive distribution of all the ICSs overlying the liver, but rather to justify the choice of the four investigated sites. Although the data may not be complete, they did not compromise the validity of recommending Site 3 (90% liver detection rate at the end-inspiratory condition, 97% at the end-expiratory condition).

### Novelty and clinical contribution

Anatomical research on ICSs has received very limited attention, possibly due to the unavailability of a standard morphometric analysis method. Since ICSs are anatomically located in subcutaneous regions, in vivo manual morphometry is practically challenging. Our study demonstrated the feasibility of utilizing US imaging to characterize multiple ICSs both morphologically and biomechanically. To our best knowledge, this study was the first to report the relevance of intercostal anatomy to the TE examination procedure. Among the four candidate sites, Site 3 is recommended as the ideal site for TE probe placement. Additionally, we propose performing TE at end-inspiration to allow for less transducer‒rib contact. It is an important advance because our finding has the potential to address operator-related dependencies and inexperience, which have hindered the broad applicability of TE. Prior to our work, site selection relied on subjective judgment and operators’ expertise, lacking standardized guidelines. Our recommendations could serve as a guide for novices and be incorporated into operational workflows to enhance examination efficiency. Moreover, these findings may be extrapolated to other liver elastography techniques, such as point ARFI or 2D-SWE, for which an adequate acoustic window is also requisite.

While most studies did not specify the exact measurement site, some have reported using somewhere between the 5th and 8th ICSs [17, 20, 21, 24]. We postulated that these choices might reflect common real-world clinical practices. Surprisingly, the reported range contradicts the existing clinical guidelines recommending probe placement in the 9th to 11th ICS [3, 7, 8]. Moreover, the consideration of a breathing technique used is also lacking in these recommendations. It was the motivation of our study that provided anatomical considerations in the context of TE. Yet, one study is rarely sufficient evidence on which to base a change. Thus, our study advocates for a more rigorous scientific evaluation of what those guidelines recommended before their widespread uptake.

### Study limitation

This study has several limitations. First, the sample size of 59 subjects enrolled appears to be relatively small. However, a *priori* analysis for the repeated measures ANOVA was performed and determined that this sample size was statistically sufficient to test our hypothesized inter-ICS differences; and it is worth noting that we have successfully demonstrated the feasibility to identify the ideal site and respiratory condition for TE. Second, our results can, at most, imply the placement of a single-element transducer, as we collected only 1D information of intercostal size (i.e., width). This anatomical information is insufficient in the context of multielement array transducers, which are commonly fabricated with a larger footprint. Future research may employ 3D imaging to characterize the course of the ICS, so as to inform an array transducer-specific placement strategy. Third, the scope of this study was confined to Chinese adults, which may raise questions about the generalizability of our findings across diverse racial demographics. Future research should encompass subjects representing varying races, geographical regions, and BMI categories. Another future research direction is to determine the relative accuracy of TE measurements obtained from different sites for staging liver fibrosis, using liver histology as the reference standard.

## Conclusion

This study observed site-dependent differences in the intercostal width, stiffness, and SCD, indicating that ICS configuration is highly variable. The data supported performing TE at Site 3 (the 8th ICS on the mid-axillary line) during the end-inspiratory phase as the measurement protocol of choice. This serves as a practical solution for challenging cases involving patients with narrow ICSs or obesity, potentially offering a universally applicable strategy without requiring technological modifications of the TE probe. Additionally, it can assist inexperienced TE practitioners and even non-specialists in shortening the learning curve. Larger studies encompassing diverse racial demographics are warranted to contextualize the findings on a global scale.

### Supplementary Information

Electronic Supplementary Material

## Data Availability

Available on request with the corresponding author.

## Abbreviations

BMI: Body mass index
ICC: Intraclass correlation coefficient
ICS: Intercostal space
SCD: Skin‒liver capsule distance
SWE: Shear-wave elastography
TE: Transient elastography
US: Ultrasound
