# Supplementary material for: Determining the ideal measurement site and respiratory condition for liver transient elastography: toward clinical practice standardization
Source: Insights Imaging. 2024 May 12;15:114. doi: 10.1186/s13244-024-01692-x (PMC11089024; doi:10.1186/s13244-024-01692-x)
Supplement: Supplementary file 1 — Electronic Supplementary Material [file 13244_2024_1692_MOESM1_ESM.pdf]

Determining the Ideal Measurement Site and Respiratory Condition for Liver Transient Elastography: Toward Clinical Practice Standardization

ELECTRONIC SUPPLEMENTARY MATERIAL

Supplementary Methods

This study adopted a within-subject design attempting to demonstrate the anatomical differences among the four measurement sites of interest and between the two respiratory conditions. To ensure the consistency of respiratory amplitudes across the conditions, the subjects wore the wearable Go Direct® chest belt (Vernier, Oregon, USA) with a built-in pressure gauge that monitored their real-time respiratory changes during the experiment.

Inter-observer and intra-observer reliability analysis. Site 1 was imaged twice by Operator 1 under the two respiratory conditions to allow for assessment of intra-observer reliability, with the probe removed from the patient and repositioned between scans. The interval was approximately 1 minute, with no change in patient condition or positioning. To establish inter-observer reliability, two operators (i.e., Operators 1 and 2) independently performed one set of intercostal acquisitions at Site 1. The first set of data collected by Operator 1 was used to assess inter-site differences.

Data processing. To minimize the measurement error, a repeated measurement strategy was implemented offline on the separate images of each captured 6-s video. For the quantification of intercostal width, three individual linear measurements using the built-in calliper were conducted in the respective frames at 2 s, 4 s, and 6 s of the video, resulting in three measurement values. The average of these three measurement values was used to represent the intercostal width of that experimental condition. For the quantification of intercostal SCD, the same methodology as outlined above was applied to measure the SCDs in the same three frames (i.e., 2 s, 4 s, 6 s). For the quantification of intercostal stiffness, the Young's moduli of the intercostal tissues were derived from the SWE images that pair with the previously measured B-mode image frames **(Figure 5)**. For each SWE image, the rater placed the largest possible circular region of interest (i.e., Q-box), avoiding bone-proximity artifacts and inclusion of subcutaneous fat within the ICS. In this fashion, the upper and lower limits of Q-box encompassed the superior border of the abdominal muscle and the inferior border of the diaphragm, respectively. The mean Young's modulus value derived from three SWE images was calculated for each experimental condition.

Statistical analyses. Operator repeatability was assessed for intercostal width, stiffness, and SCD measurements using intra-class correlation coefficients (ICCs). The repeatability was classified based on ICC values as follows: poor (ICC:0–0.5), fair/moderate (ICC:0.5–0.75), good (ICC:0.75–0.9), and excellent (ICC:0.81-1.00) (1).To evaluate the demographic and anthropometric factors that possibly influenced the width and stiffness of the ICS, we performed univariate analysis using Spearman's correlation test for continuous candidate variables, and the Mann–Whitney U test for categorical candidate variables. Variables that achieved statistical significance (p <0.05) in univariate analysis were included in a multivariate model. The multiple regression model was constructed with age, height, weight, BMI, SCD, waist circumference, sex, and metabolic syndrome as candidate covariates, and the width and stiffness as the outcome variables.

<u>Supplementary Results</u>

Subject characteristics. Overall, the median age was 57 years (IQR: 43-64; range: 27-71) with 51% females. The median BMI was 24 kg/m^2 (IQR: 21-26; range: 17- 39); 27% and 7% of patients were classified as overweight (BMI≥25 kg/m^2) and obese (BMI≥30 kg/m^2), respectively. The majority (78%) were experiencing chronic hepatitis B (32% with coexistent steatosis) and 22% had non-alcoholic fatty liver disease (NAFLD).

Reliability of US measurement. Of the 59 subjects, 34 participated in the intra-operator reliability analysis, while 24 were assessed for the inter-operator reliability **(Supplementary Table 1)**. For the intra-operator reliability of width and SCD measurements, the ICCs$_{(3,3)}$ of end-inspiratory and end-expiratory conditions ranged between 0.972 and 0.992. Additionally, all ICCs$_{(2,3)}$ for inter-operator reliability were greater than 0.97. Both measures reflected excellent observer reliability. In comparison, inter- and intra-observer reliability for intercostal stiffness measurements were fair to good, with higher intra-operator ICC$_{(3,3)}$ (between 0.790 and 0.896) than inter-operator ICC$_{(2,3)}$ (between 0.593 and 0.683). This may imply the impact of operator experience, as the operator gained experience across two successive scans in an intra-operator setting. The lowest ICC$_{(2,3)}$ of 0.593 (95% CI: 0.060–0.824) was noted in the inter-observer reliability at end-inspiration.

Influencing factors on intercostal width and stiffness. Supplementary Table 2 and **Supplementary Table 3** show the results of both univariate and multivariate analyses evaluating the factors associated with the 16 experimental conditions. In univariate analysis **(Supplementary Table 2)**, greater weight, higher BMI, and larger waist circumference were strongly associated with larger width values (p <0.001 in all conditions). Height was positively correlated to the width of the ICS at Sites 1 to 3, but not at Site 4 (end-inspiratory condition: p =0.140, end-expiration condition: p =0.201). An increase in SCD was fairly

associated with an increase in the width of the ICS at all sites (r_s = 0.27-0.35). Wider ICSs were more likely to be identified in patients with metabolic syndrome (Site 1, end-expiratory condition: p = 0.012; Site 2, end-inspiratory condition: p = 0.002; Site 3, end-expiratory condition: p = 0.027; Site 4, end-inspiratory and end-expiratory conditions: p <0.001 and p = 0.003). There was a significant influence of gender on width values, with males having wider ICSs except for Site 3 (end-inspiratory condition: p = 0.138, end-expiratory condition: p = 0.124). Whereas no association between age and width was seen, older age fairly correlated with greater stiffness at one site (Site 3: r_s = 0.37, p = 0.004). With the exception of height (Site 3: r_s = -0.30, p = 0.021), none were significant predictors of the stiffness of the ICS.

In multivariate analysis **(Supplementary Table 3)**, height, weight, BMI, SCD, gender, and the presence of metabolic syndrome remained independent factors associated with intercostal width. Waist circumference no longer provided a significant contribution to the width values in this model. The beta weights showed that weight (Site 1, end-inspiratory condition: β = -2.91, p = 0.041) had the greatest influence on the regression model, followed by BMI (Site 1, end-inspiratory condition: β = 2.75, p = 0.011; end-expiratory condition: β = 2.47, p = 0.018). However, only SCD and BMI were independently associated with the width values at most sites and appeared to be the most general determinants. The only covariate influencing the stiffness values was height (β = -2.03; p = 0.029), which was in line with the result of univariate analysis.

Supplementary Discussion

Measurement reliability. Excellent intra- and inter-operator agreement of the morphological measurements of width and SCD were observed, with all ICCs exceeding 0.97. In contrast, the measures of intercostal stiffness were less reliable, with ICCs ranging between 0.593 and 0.896. This reduction in ICCs may be attributed to SWE signal interference from adjacent ribs. A previous study (2) also reported the relatively low repeatability for SWE of the diaphragm ($ICC_{(3,1)}$ = 0.68) and intercostal muscles ($ICC_{(3,1)}$ = 0.44). Inter-operator $ICCs_{(2,3)}$ of intercostal SWE was found to be lower than intra-operator $ICCs_{(3,3)}$. We postulated that this variation may be due to discrepancies in the actual measurement site or differences in probe pressures applied between operators. It is worth noting that we attempted to minimize this measurement error by marking the investigated sites with a surgical skin marker to achieve higher repeatability. Placing the probe with minimal pressure was also required to prevent deformation of the intercostal tissues being examined. Despite these efforts, there is still room for improvement in terms of standardizing probe positioning to ensure reliable intercostal US measurements.

Influencing factors and implication for patient selection. Height was the only predictor of intercostal stiffness. Intercostal width, on the other hand, varied considerably depending on gender, BMI, and SCD. Only SCD had a homogeneous influence on the width across all sites, suggesting that subcutaneous fat accumulation contributes to a wider ICS. We also confirmed narrower ICSs in women which aligns with a previous study (3). Our findings have clinical implications for patient selection and stratification based on patient characteristics: taller males with greater SCD and BMI tend to favour TE probe placement. However, a dilemma arises, as the risk of TE failure might increase in this population due to the associated overmuch fat layers. Consequently, hepatologists are strongly urged to exercise caution when applying these regression results in pre-TE planning.

Supplementary References

1. Koo TK, Li MY. A Guideline of Selecting and Reporting Intraclass Correlation Coefficients for Reliability Research. JOURNAL OF CHIROPRACTIC MEDICINE 2016;15(2):155-163. doi: 10.1016/j.jcm.2016.02.012
2. Pałac M, Linek P. Intra-Rater Reliability of Shear Wave Elastography for the Quantification of Respiratory Muscles in Adolescent Athletes. Sensors (Basel, Switzerland) 2022;22(17):6622. doi: 10.3390/s22176622
3. Kim Y-S, Park MJ, Rhim H, Lee MW, Lim HK. Sonographic analysis of the intercostal spaces for the application of high-intensity focused ultrasound therapy to the liver. American journal of roentgenology (1976) 2014;203(1):201-208. doi: 10.2214/AJR.13.11744

Supplementary Table 1. Intra- and inter-operator reliability results for US measurements of the intercostal space.

	Intra-operator reliability (n=34)		Inter-operator reliability (n=24)	
**Intercostal US measurement**	**$ICC_{(3,3)}$[a]**	**95% confidence interval**	**$ICC_{(2,3)}$[b]**	**95% confidence interval**
Width at end-inspiration (mm)	0.981	(0.963 – 0.991)	0.978	(0.950 – 0.991)
Width at end-expiration (mm)	0.986	(0.972 – 0.993)	0.977	(0.948 – 0.990)
SCD at end-inspiration (mm)	0.992	(0.985 – 0.996)	0.976	(0.944 – 0.989)
SCD at end-expiration (mm)	0.972	(0.943 – 0.986)	0.986	(0.969 – 0.994)
Stiffness at end-inspiration (kPa)	0.896	(0.791 – 0.948)	0.593	(0.060 – 0.824)
Stiffness at end-expiration (kPa)	0.790	(0.580 – 0.895)	0.683	(0.268 – 0.863)

ICC = intraclass correlation coefficient.

[a]ICC computed using two-way mixed model and consistency; [b]ICC computed using two-way random model and consistency.

Supplementary Table 2. Factors associated with the width and stiffness of the intercostal space in univariate analyses (n=59).

		Age		Height		Weight		BMI		SCD		Waist circumference		Sex		Metabolic syndrome	
		r_s	*p*	r_s	*p*	r_s	*p*	r_s	*p*	r_s	*p*	r_s	*p*	Mean ± SD	*p*	Mean ± SD	*p*
**Width (mm)**																	
Site 1	Inspiration	-	0.478	-	0.085	0.485	**<0.001***	0.514	**<0.001***	0.269	**0.039***	0.563	**<0.001***	Male, 17.0±4.1; Female, 15.6±3.8	0.197	Absent, 16.0±3.9; Present, 18.5±4.0	0.128
	Expiration	-	0.496	0.301	**0.021***	0.545	**<0.001***	0.523	**<0.001***	0.297	**0.022***	0.601	**<0.001***	Male, 15.2±4.2; Female, 13.2±3.6	**0.050***	Absent, 13.7±3.7; Present, 17.7±4.5	**0.012***
Site 2	Inspiration	-	0.561	0.275	**0.035***	0.526	**<0.001***	0.544	**<0.001***	0.266	**0.042***	0.532	**<0.001***	Male, 20.1±4.1; Female, 17.8±3.1	**0.019***	Absent, 18.4±3.5; Present, 22.9±3.7	**0.002***
	Expiration	-	0.774	-	0.174	0.482	**<0.001***	0.529	**<0.001***	-	0.101	0.510	**<0.001***	Male, 18.7±4.2; Female, 17.2±7.5	0.370	Absent, 17.5±6.2; Present, 21.1±3.6	0.141
Site 3	Inspiration	-	0.267	0.272	**0.037***	0.551	**<0.001***	0.585	**<0.001***	0.314	**0.015***	0.563	**<0.001***	Male, 20.0±4.5; Female, 18.1±5.1	0.139	Absent, 18.7±4.9; Present, 22.0±3.6	0.089
	Expiration	-	0.199	0.259	**0.048***	0.584	**<0.001***	0.632	**<0.001***	0.354	**0.006***	0.580	**<0.001***	Male, 17.6±4.3; Female, 15.7±4.9	0.124	Absent, 16.1±4.6; Present, 20.2±3.5	**0.027***
Site 4	Inspiration	-	0.711	-	0.140	0.418	**<0.001***	0.454	**<0.001***	-	0.067	0.489	**<0.001***	Male, 17.8±3.4; Female, 15.7±3.4	**0.019***	Absent, 16.1±3.2; Present, 20.8±2.9	**<0.001***
	Expiration	-	0.941	-	0.201	0.466	**<0.001***	0.540	**<0.001***	0.296	**0.023***	0.526	**<0.001***	Male, 15.8±3.6; Female, 13.4±3.3	**0.010***	Absent, 14.1±3.4; Present, 18.3±3.4	**0.003***
**Stiffness (kPa)**																	
Site 1	Inspiration	-	0.876	-	0.973	-	0.364	-	0.380	-	0.892	-	0.323	Male, 17.5±7.7; Female, 17.7±9.3	0.779	Absent, 17.7±9.0; Present, 17.3±2.7	0.542
	Expiration	-	0.465	-	0.389	-	0.716	-	0.428	-	0.282	-	0.365	Male, 14.1±3.6; Female, 17.0±9.1	0.481	Absent, 15.6±7.5; Present, 15.3±3.0	0.573
Site 2	Inspiration	-	0.646	-	0.657	-	0.843	-	0.386	-	0.710	-	0.865	Male, 15.6±8.0; Female, 15.6±9.0	0.628	Absent, 15.7±8.7; Present, 14.8±6.8	0.954
	Expiration	-	0.164	-	0.675	-	0.305	-	0.114	-	0.055	-	0.409	Male, 14.0±7.5; Female, 12.4±5.3	0.539	Absent, 13.0±6.6; Present, 14.4±5.4	0.441
Site 3	Inspiration	-	0.646	-	0.200	-	0.692	-	0.733	-	0.860	-	0.956	Male, 13.0±5.7; Female, 16.1±7.2	0.065	Absent, 14.2±5.8; Present, 17.6±11.4	0.705
	Expiration	0.371	**0.004***	-0.299	**0.021***	-	0.301	-	0.876	-	0.503	-	0.773	Male, 11.8±8.0; Female, 13.2±5.3	0.057	Absent, 12.5±7.1; Present, 12.9±3.8	0.413
Site 4	Inspiration	-	0.633	-	0.396	-	0.480	-	0.375	-	0.671	-	0.861	Male, 11.7±4.8; Female, 14.7±7.1	0.072	Absent, 12.8±5.3; Present, 16.4±11.0	0.441
	Expiration	-	0.821	-	0.394	-	0.930	-	0.737	-	0.782	-	0.785	Male, 10.2±5.0; Female, 12.2±6.8	0.246	Absent, 11.0±5.7; Present, 13.7±8.3	0.441

Note: age, height, weight, BMI, SCD and waist circumference were analysed by Spearman's correlation test; sex and metabolic syndrome were analysed by unpaired t-test or Mann-Whitney U test, where appropriate.
* $p \leq 0.05$; BMI = body mass index; SCD = skin-liver capsule distance; ICS = intercostal space; r_s = Spearman's rank correlation coefficient.

Supplementary Table 3. Factors associated with the width and stiffness of the intercostal space in multivariate analyses.

		Age		Height		Weight		BMI		SCD		Waist circumference		Sex		Metabolic syndrome	
		β	*p*	β	*p*	β	*p*	β	*p*	β	*p*	β	*p*	β	*p*	β	*p*
**Width (mm)**																	
Site 1	Inspiration	-0.040	0.752	1.556	**0.047***	-2.909	**0.041***	2.753	**0.011***	-0.441	**0.026***	0.310	0.324	0.005	0.980	0.090	0.513
	Expiration	-0.082	0.502	1.423	0.062	-2.576	0.062	2.471	**0.018***	-0.389	**0.042***	0.232	0.448	-0.049	0.783	0.228	0.093
Site 2	Inspiration	0.011	0.927	0.513	0.466	-0.919	0.471	1.284	0.166	-0.579	**0.004***	0.301	0.336	-0.068	0.709	0.264	**0.041***
	Expiration	0.102	0.495	-0.813	0.354	1.150	0.467	-0.387	0.735	-0.338	0.160	0.013	0.974	-0.171	0.448	0.149	0.347
Site 3	Inspiration	-0.076	0.540	0.854	0.245	-1.332	0.317	1.904	0.059	-0.693	**0.002***	0.172	0.574	0.073	0.679	0.095	0.459
	Expiration	-0.070	0.541	1.101	0.109	-1.809	0.146	2.391	**0.012***	-0.668	**0.001***	0.037	0.895	0.028	0.862	0.181	0.132
Site 4	Inspiration	-0.025	0.829	0.354	0.592	-1.008	0.401	1.462	0.095	-0.705	**0.001***	0.280	0.356	-0.310	0.065	0.322	**0.009***
	Expiration	-0.023	0.838	0.478	0.449	-1.437	0.211	1.897	**0.025***	-0.676	**0.001***	0.180	0.532	-0.457	**0.005***	0.275	**0.019***
**Stiffness (kPa)**																	
Site 1	Inspiration	-0.025	0.871	-0.007	0.994	-0.245	0.887	0.820	0.524	-0.132	0.578	-0.386	0.317	-0.150	0.507	0.049	0.770
	Expiration	0.059	0.688	-2.030	**0.029***	3.282	0.051	-2.225	0.076	0.315	0.170	-0.334	0.368	-0.075	0.728	-0.032	0.845
Site 2	Inspiration	-0.023	0.885	0.024	0.980	-0.277	0.871	0.053	0.965	0.180	0.484	-0.009	0.982	-0.134	0.583	-0.037	0.826
	Expiration	0.109	0.471	0.328	0.709	-1.324	0.407	1.116	0.335	-0.177	0.461	-0.063	0.872	-0.430	0.063	0.115	0.469
Site 3	Inspiration	0.106	0.467	0.735	0.397	-1.505	0.339	1.139	0.334	0.404	0.107	-0.244	0.501	0.101	0.627	0.205	0.179
	Expiration	0.248	0.090	0.356	0.677	-1.656	0.287	1.419	0.224	0.029	0.905	0.047	0.896	-0.326	0.117	-0.030	0.841
Site 4	Inspiration	0.049	0.749	0.361	0.679	-0.827	0.600	0.349	0.759	0.241	0.366	0.238	0.552	0.264	0.229	0.136	0.390
	Expiration	0.079	0.615	-0.287	0.745	0.182	0.909	0.164	0.887	0.204	0.450	-0.313	0.440	-0.008	0.972	0.179	0.265

Note: age, height, weight, BMI, SCD, waist circumference, sex and metabolic syndrome were analysed by multiple linear regression.

* $p \le 0.05$; BMI = body mass index; SCD = skin-liver capsule distance; ICS = intercostal space; β = standardized regression coefficient.
